# Prevalence and predictors of irritable bowel syndrome in patients with morbid obesity: a cross-sectional study

**DOI:** 10.1186/s40608-017-0159-z

**Published:** 2017-06-29

**Authors:** Martin Aasbrenn, Ingvild Høgestøl, Inger Eribe, Jon Kristinsson, Stian Lydersen, Tom Mala, Per G. Farup

**Affiliations:** 1 Department of Surgery, Innlandet Hospital Trust, Kyrre Grepps gate 11, N-2819 Gjøvik, Norway; 2 Unit for Applied Clinical Research, Department of Cancer Research and Molecular Medicine, Faculty of Medicine and Health Sciences, Norwegian University of Science and Technology, Trondheim, Norway; 3 Department of Endocrinology, Morbid Obesity and Preventive Medicine, Oslo University Hospital, Oslo, Norway; 4 Institute of Clinical Medicine, Faculty of Medicine, University of Oslo, Oslo, Norway; 5 Regional Centre for Child and Youth Mental Health and Child Welfare, Faculty of Medicine and Health Sciences, Norwegian University of Science and Technology, Trondheim, Norway; 6 Department of Research, Innlandet Hospital Trust, Brumunddal, Norway

**Keywords:** Irritable bowel syndrome, Functional bowel disorders, Morbid obesity, Abdominal pain, Functional gastrointestinal disorders, Low-density lipoprotein

## Abstract

**Background:**

Irritable bowel syndrome has been reported as more common in patients with morbid obesity than in the general population. The reason for this association is unknown. The aims of this study were to study the prevalence of irritable bowel syndrome and other functional bowel disorders in patients with morbid obesity, and to search for predictors of irritable bowel syndrome.

**Methods:**

Patients opting for bariatric surgery at two obesity centers in South-Eastern Norway were included. Functional bowel disorders were diagnosed according to the Rome III criteria. Predictors were evaluated in a multivariable logistic regression analysis with irritable bowel syndrome as the dependent variable.

**Results:**

A total of 350 (58%) out of 603 consecutive patients were included. The prevalence rates of irritable bowel syndrome at the two centers were 17/211 (8%) and 37/139 (27%) respectively. High low-density lipoprotein (OR 2.10; 95% CI 1.34–3.29), self-reported psychiatric disorders (OR 2.39; 95% CI 1.12–5.08) and center (OR 5.22; 95% CI 2.48–10.99) were independent predictors of irritable bowel syndrome.

**Conclusions:**

At one of the two obesity centers, the prevalence of irritable bowel syndrome was threefold higher than in the general population in the same region. The high prevalence appears to be related to dietary differences or altered absorption or metabolism of fat. Attention to irritable bowel syndrome is important in the care of patients with morbid obesity.

## Background

Irritable bowel syndrome (IBS) has a prevalence of about 7% in North America and Europe [1]. Abdominal pain or discomfort is the main symptom [2]. The pathophysiology includes disturbances of the gut-brain axis, low-grade mucosal immune activation and changes in the fecal microbiota [3, 4]. Because no biomarker is available, the gold standard for the diagnosis is symptom-based criteria [2, 5, 6]. IBS is more prevalent in women than in men and is associated with several comorbid conditions including anxiety and depression [7].

Most reports indicate that IBS is more common in patients with morbid obesity than in the general population, with prevalence rates from 8 to 31% in small series [8–12]. The reason for this association is unknown [13]. Pathophysiological factors that are common for IBS and MO, including psychological distress, low-grade systemic inflammation and vitamin deficiencies, could explain the association [3, 14–19]. Insights into the risk factors of IBS among patients with morbid obesity might help to prevent this burdensome condition in patients with obesity, and improve our knowledge of the general pathophysiology of IBS.

The aims of this study were to explore the prevalence of IBS, subtypes of IBS and other functional bowel disorders and to search for predictors of IBS in two groups of patients with morbid obesity.

## Methods

### Study design and setting

In this cross-sectional study, adult patients referred to two obesity centers providing bariatric surgery in South-Eastern Norway were invited to participate. Oslo University Hospital Aker (OUH-A) recruited patients living in an urban area and Innlandet Hospital Trust Gjøvik (IHT-G) recruited patients living in rural areas and small towns. The medical history, current medications and anthropometric evaluations including BMI were registered on the day of inclusion. A routine clinical examination was performed and blood samples were retrieved. Demographics and comorbidity were reported by the patients in a paper-based case report form. All patients filled in questionnaires for the classification of functional bowel disorders. Additional diagnostic procedures including endoscopic examinations were done at the discretion of the attending physician. Patients at OUH-A and IHT-G were recruited from February 2014 through April 2015, and from December 2012 through September 2014, respectively.

### Participants

The inclusion criteria were age 18–65 years and morbid obesity, defined as BMI > 40 kg/m^2^ or BMI >35 kg/m^2^ with obesity-related comorbidity at the time of referral [20]. Exclusion criteria were major psychiatric disorders (schizophrenia, major depression or bipolar disorder), alcohol and drug addiction, organic gastrointestinal disorders, former obesity surgery and other major abdominal surgery. The case report form was printed in Norwegian, and patients not able to understand Norwegian were excluded. At IHT-G, patients were included only 3 days per week when the study nurse was present.

### Variables

#### Demographics

Seven demographic variables were registered: Age (years), sex (male/female), ethnicity (% Caucasian), BMI (kg/m^2^), smoking habits (smoking/not smoking), work status (full-time/part-time/not working) and cohabitant status (living with partner/not living with partner).

#### Comorbidity and use of medication

Six present or previous comorbidities were reported by the patient on the case report form: Diabetes mellitus, hypothyroidism, hypertension, fibromyalgia, gallstones and self-reported psychiatric disorders. At OUH-A, the subjects were asked if they had been diagnosed with anxiety or depression (present/absent), and at IHT-G if they had sought professional help for psychiatric disorders (present/absent). At both centres, subjects with a diagnosis of major psychiatric disorders (schizophrenia, major depression or bipolar disorder) were excluded. When in doubt, the subjects were referred for a psychiatric evaluation. Regular use of medication was reported by the patients. All information concerning comorbidity and medication were reviewed by a clinician with full access to the patient’s medical record.

#### Abdominal complaints

Functional bowel disorders were diagnosed with a validated Norwegian translation of the Rome III questionnaire [2]. IBS and subtypes of IBS, functional constipation, functional diarrhea and functional bloating were coded as present/absent.

#### Blood tests

Thirty-tree variables were analyzed from the blood samples. The reference values for the 15 variables reported in the results were as follows: hemoglobin g/dl: women 11.7–15.3, men 13.4–17.0; white-cell count 10^9^/l: 3.5–10.0; platelet count 10^9^/l: 145–390; c-reactive protein (CRP) mg/l: <5; cholesterol mmol/l: age 18–29 2.9–6.1, age 30–49 3.3–6.9, age > 50 3.9–7.8; high-density lipoprotein mmol/l: women 1.0–2.7, men 0.8–2.1; low-density lipoprotein (LDL) mmol/l: age 18–29 1.3–4.3, age 30–49 1.5–4.8, age > 50 2.0–5.4; thyroid stimulating hormone (TSH) mIE/l: 0.27–4.20; free thyroxin (T_4_) pmol/l: 8.0–22.0; vitamin B_1_ nmol/l: 95–200; vitamin B_6_ nmol/l: 15–160; vitamin B_12_ pmol/l: 140–650; folic acid nmol/l: 7–40; HbA_1_C %: 4.0–6.0; total bilirubin μmol/l: 5–25. The other 18 variables were mean corpuscular volume, mean corpuscular hemoglobin, iron, transferrin, transferrin saturation, ferritin, transferrin iron binding capacity, sodium, potassium, magnesium, phosphate, glucose, creatinine, uric acid, alanine aminotransferase, total protein, albumin, and triglycerides.

#### Dietary registration

At IHT-G, the intake of micro- and macronutrients was estimated with a semi-quantitative food frequency questionnaire designed and validated for the Norwegian population [21].

### Statistical analysis

Data are presented as mean (standard deviation), median (range) and proportion (percentage) according to the distribution of data. Student’s t-test, Mann-Whitney U test, Pearson chi-squared test, or Fisher’s exact test was used for the comparisons between the groups depending on the type of data and normality. Correlations were assessed with the Pearson or Spearman correlation coefficients. Because the prevalence of IBS differed between the centers, the predictors of IBS were analyzed one-by-one with logistic regression adjusted for the center after testing for statistical interaction. The effect of TSH differed strongly from linear. Therefore, fractional polynomials were used to transform this variable. Predictors that were significant in these analyses were included as independent variables in a multivariable logistic regression analysis with IBS as the dependent variable. The results are presented as odds ratios with 95% confidence intervals (CI). The presented predictors of IBS include all predictors that were significant in the analysis corrected for center only and a selection of other relevant variables. In the posthoc analysis, we examined the differences between the groups of patients with and without IBS separately at the two centers. Two-sided *p*-values <0.05 were judged to indicate statistical significance. Data analysis was performed with IBM SPSS Statistics for Windows, Version 21.0 (Armonk, NY: IBM Corp) and Stata Statistical Software, Release 13 (College Station, TX: StataCorp LP).

### Power calculation

The prevalence of IBS was 8.4% in the general population from the same area [14] and was expected to be 18% in patients with morbid obesity [9]. A study including 350 participants with morbid obesity was calculated to have a power of 98% to detect a difference between the general population and the patients with morbid obesity, with α = 0.01.

### Ethics

The study was approved by the Regional Committee for Medical and Health Research Ethics South East Norway, references 2012/966 and 2013/1264, and conducted in accordance with the Declaration of Helsinki. Written informed consent was obtained from all individual participants included in the study.

## Results

**Fig. 1:**
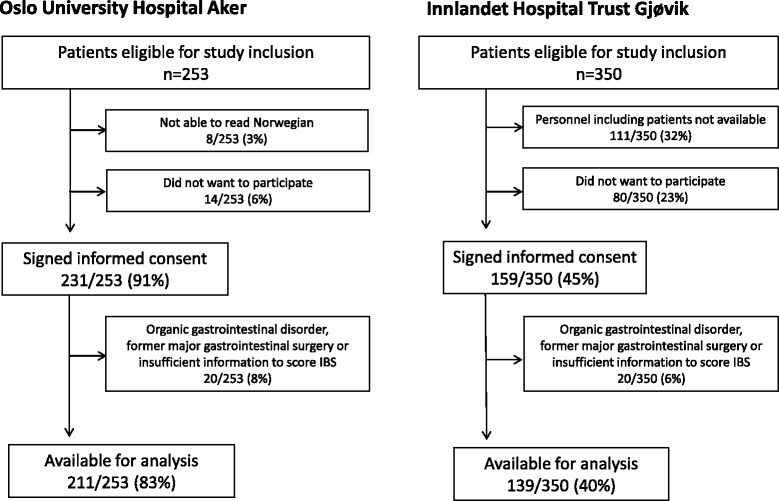
Flow chart depicting inclusion of patients at the two obesity centres

A total of 350 (58%) out of 603 consecutive patients eligible for study participation were included (Fig. 1). The prevalence rates of IBS were 17/211 (8%) at OUH-A and 37/139 (27%) at IHT-G (*p* < 0.001), and the prevalence rates of functional constipation were 20/205 (10%) and 3/135 (2%) respectively (*p* = 0.006). Table 1 gives patients’ characteristics at the two centers with comparisons between the groups.

**Table 1:** Patients’ characteristics at the two centers

	Oslo University Hospital Aker		Innlandet Hospital Trust Gjøvik		
		n		n	*p*-value
Gender (%male)	62 (29%)	211	28 (20%)	139	0.05^#^
Age (years)	43 (21–61)	211	44 (24–61)	139	0.66*
Body mass index (kg/m^2^)	43 (33–62)	211	42 (35–53)	139	0.09*
Ethnicity (% Caucasian)	195 (93%)	210	139 (100%)	139	0.001^#^
Irritable bowel syndrome (IBS)	17/211 (8%)	211	37/139 (26%)	139	<0.001^#^
IBS-constipation	3/17 (18%)	17	7/37 (19%)	37	0.48^#^
IBS-diarrhea	2/17 (12%)	17	11/37 (30%)	37	
IBS-mixed	11/17 (65%)	17	18/37 (49%)	37	
IBS-unsubtyped	1/17 (6%)	17	1/37 (3%)	37	
Functional bloating	21/205 (10%)	205	19/138 (14%)	138	0.32^#^
Functional constipation	20/205 (10%)	205	3/135 (2%)	138	0.006^#^
Functional diarrhea	9/206 (4%)	206	4/139 (3%)	139	0.48^#^
Smoking	56/204 (28%)	204	24/139 (17%)	139	0.03^#^
Diabetes mellitus	53/211 (25%)	211	26/139 (19%)	139	0.16^#^
Hypothyroidism	19/211 (9%)	211	17/138 (12%)	138	0.32^#^
Fibromyalgia	20/211 (10%)	211	25/138 (18%)	138	0.02^#^
Self-reported psychiatric disorder	36/211 (17%)	211	29/139 (21%)	139	0.37^#^
Hemoglobin (g/dl)	14.1 (1.1)	211	14.5 (1.1)	136	0.006
White-cell count (×10^9^/l)	7.4 (2.4–13.3)	210	7.5 (4.2–16.1)	136	0.95*
Platelet count (×10^9^/l)	275 (112–519)	210	276 (131–939)	134	0.46*
HbA_1_C (%)	5.8 (4.7–14.9)	211	5.5 (4.5–11.5)	136	<0.001*
Bilirubin (μmol/l)	7 (2–23)	210	6 (2–28)	136	0.07*
C-reactive protein (mg/l)	7 (1–50)	210	5 (0–43)	136	0.01*
Cholesterol (mmol/l)	4.9 (1.0)	210	5.1 (1.0)	136	0.10
High-density lipoprotein (mmol/l)	1.1 (0.5–2.0)	210	1.1 (0.4–2.2)	136	0.70*
Low-density lipoprotein (mmol/l)	3.1 (0.9)	210	3.3 (0.9)	136	0.02
Thyroid stimulating hormone (mIE/l)	1.4 (0–14.6)	210	1.7 (0–6.9)	136	0.11*
Free T_4_ (pmol/l)	15 (11–28)	210	15 (10–26)	136	0.30*
Vitamin B_1_ (nmol/l)	156 (62–239)	210	153 (104–246)	135	0.70*
Vitamin B_6_ (nmol/l)	29 (5–231)	211	22 (5–209)	133	0.01*
Vitamin B_12_ (pmol/l)	344 (158–1480)	211	346 (158–1401)	136	0.80*
Folic acid (nmol/l)	18 (6–46)	211	17 (7–46)	136	0.14*
Use of statins	35/211 (17%)	211	19/138 (14%)	138	0.48^#^
Use of thyroid substitution therapy	19/211 (9%)	211	13/139 (9%)	139	0.91^#^

The results are given as number (proportion in percent) for categorical variables, mean (standard deviation) for continuous variables with normal distribution and median (range) for other continuous variables. Data were analyzed with with t-tests, Pearson chi-squared tests (marked with ^#^) or Mann-Whitney U test (marked with *)

High serum levels of LDL, self-reported psychiatric disorders and center were independent predictors of IBS (Table 2). Table 3 gives the comparisons between patients with and without IBS at each center, and comparisons between patients with IBS at the two centers. TSH was higher, and free T_4_ was lower among the IBS patients at IHT-G compared to those at OUH-A. LDL levels in the blood correlated with higher relative energy intake from saturated fat (*r* = 0.26, *p* = 0.01) and monounsaturated fat (*r* = 0.25, *p* = 0.01). Patients with IBS had lower relative energy intake from proteins. No other significant differences in nutrition between patients with and without IBS were observed (Table 4).

**Table 2:** Predictors of irritable bowel syndrome (IBS)

			Adjusted for center only		Adjusted for all significant predictors	
	Patients without IBS	Patients with IBS	Odds ratio (95% CI)	*p*-value	Odds ratio (95% CI)	*p*-value
Gender (%male)	84 (28%)	6 (11%)	0.35 (0.14–0.87)	0.02	0.57 (0.18–1.82)	0.34
Age (years)	44 (21–61)	41 (23–61)	0.97 (0.94–1.00)	0.07		
Body mass index (kg/m^2^)	42 (33–62)	42 (36–53)	0.98 (0.92–1.06)	0.64		
Ethnicity (% Caucasian)	281/296 (95%)	53/54 (98%)	1.34 (0.17–10.82)	0.51		
Smoking	68/291 (23%)	12/52 (23%)	1.24 (0.60–2.59)	0.56		
Diabetes mellitus	72/296 (24%)	7/54 (13%)	0.51 (0.22–1.20)	0.12		
Hypothyroidism	29/296 (10%)	7/53 (13%)	1.27 (0.51–3.16)	0.61		
Fibromyalgia	33/295 (11%)	12/54 (22%)	1.86 (0.86–4.00)	0.12		
Self-reported psychiatric disorder	47/296 (16%)	18/54 (33%)	2.61 (1.33–5.13)	0.005	2.39 (1.12–5.08)	0.02
Hemoglobin (g/dl)	14.3 (1.1)	14.0 (1.1)	0.65 (0.49–0.88)	0.004	0.68 (0.45–1.02)	0.07
White-cell count (×10^9^/l)	7.5 (2.8–16.1)	7.3 (2.4–11.3)	0.85 (0.72–1.00)	0.05		
Platelet count (×10^9^/l)	275 (112–519)	294 (131–939)	1.00 (1.00–1.01)	0.14		
C-reactive protein (mg/l)	6 (0–50)	5 (1–23)	0.97 (0.92–1.03)	0.33		
Cholesterol (mmol/l)	4.9 (1.0)	5.4 (0.8)	1.73 (1.23–2.43)	0.002		
High-density lipoprotein (mmol/l)	1.1 (0.4–2.2)	1.1 (0.7–2.0)	1.02 (0.39–2.67)	0.97		
Low-density lipoprotein (mmol/l)	3.1 (0.9)	3.6 (0.7)	1.85 (1.27–2.70)	0.001	2.10 (1.34–3.29)	0.001
Thyroid stimulating hormone (mIE/l)	1.5 (0.0–7.9)	1.7 (0.2–14.6)		0.02		0.08
Free T_4_ (pmol/l)	15 (10–28)	15 (11–23)	0.97 (0.85–1.10)	0.17		
Vitamin B_1_ (nmol/l)	155 (62–239)	142 (91–246)	0.98 (0.97–1.00)	0.008	0.99 (0.98–1.01)	0.21
Vitamin B_6_ (nmol/l)	26 (5–231)	26 (6–113)	0.99 (0.98–1.01)	0.36		
Vitamin B_12_ (pmol/l)	346 (158–1480)	342 (173–712)	1.00 (1.00–1.00)	0.52		
Folic acid (nmol/l)	18 (6–46)	16 (7–46)	0.98 (0.94–1.02)	0.28		
Use of statins	52/295 (18%)	2/54 (4%)	0.18 (0.04–0.78)	0.02	0.31 (0.07–1.47)	0.14
Use of thyroid substitution therapy	26/296 (9%)	6/54 (11%)	1.30 (0.49–3.46)	0.60		
Center					5.22 (2.48–10.99)	<0.001

The results are given as number (proportion in percent) for categorical variables, mean (standard deviation) for continuous variables with normal distribution and median (range) for other continuous variables. In the column “Adjusted for center only”, comparisons between patients with and without IBS were performed with logistic regression adjusted for center. In total, 48 potential predictors were examined. The 25 predictors presented in the tables are all predictors with significant associations with IBS and a selection of other potential predictors of clinical interest. In the column “Adjusted for all significant predictors”, gender, self-reported psychiatric disorders, hemoglobin, low-density lipoprotein, thyroid stimulating hormone, vitamin B_1_, use of statins and center were included in the final logistic regression analysis with 342 patients. Cholesterol is not included in the final analysis due to high correlation with LDL. No odds ratio for thyroid stimulating hormone is available as the variable is transformed with fractional polynomials

**Table 3:** Comparisons between patients with and without irritable bowel syndrome (IBS) at the two centers

	Oslo University Hospital Aker			Innlandet Hospital Trust Gjøvik			
	No IBS *n* = 194	IBS *n* = 17	*p*-value	No IBS *n* = 102	IBS *n* = 37	*p*-value	Differences between patients with IBS at the two centers (*p*-values)
Fibromyalgia	19/194 (10%)	1/17 (6%)	0.71^##^	14/101 (14%)	11/37 (30%)	0.03^#^	0.08^##^
Self-reported psychiatric disorder	30/194 (16%)	6/17 (35%)	0.04^#^	17/102 (17%)	12/37 (32%)	0.04^#^	0.84^#^
Hemoglobin (g/dl)	14.2 (1.1)	13.8 (1.1)	0.23	14.6 (1.0)	14.0 (1.1)	0.006	0.53
HbA_1_C (%)	5.8 (4.7–14.9)	5.5 (4.9–7.3)	0.02*	5.4 (4.5–11.5)	5.5 (4.9–9.7)	0.52*	0.94*
Bilirubin (μmol/l)	7 (2–23)	7 (4–19)	0.94*	7 (2–28)	5 (2–15)	0.03*	0.07*
Cholesterol (mmol/l)	4.9 (1.0)	5.1 (0.8)	0.44	4.9 (1.0)	5.5 (0.8)	0.001	0.05
Low-density lipoprotein (mmol/l)	3.1 (0.9)	3.3 (0.7)	0.26	3.2 (0.9)	3.7 (0.7)	0.001	0.07
Thyroid stimulating hormone (mU/l)	1.5 (0.0–7.9)	1.4 (0.2–14.6)	0.31*	1.5 (0.0–5.5)	2.1 (0.8–6.9)	0.001*	0.007*
Free T_4_ (pmol/l)	15 (11–28)	16 (12–23)	0.14*	16 (10–26)	15 (11–21)	0.04*	0.02*
Vitamin B_1_ (nmol/l)	157 (62–239)	145 (91–176)	0.03*	155 (104–232)	142 (112–246)	0.04*	0.58*
Use of statins	34/194 (18%)	1/17 (6%)	0.32^##^	18/101 (18%)	1/37 (3%)	0.02^#^	0.54^##^

The results are given as number (proportion in percent) for categorical variables, mean (SD) for continuous variables with normal distribution and median (range) for other continuous variables. All the predictors that showed significant associations with IBS (*p* < 0.05) at one of the two centers are shown in the table. The differences between patients with and without IBS at Oslo University Hospital Aker, between patients with and without IBS at Innlandet Hospital Trust Gjøvik and between patients with IBS at Innlandet Hospital Trust Gjøvik and patients with IBS at Oslo University Hospital Aker are analyzed with t-tests, Pearson chi-squared tests (marked with ^#^), Mann-Whitney U test (marked with *) or Fisher’s exact test (marked with ^##^)

**Table 4:** Diet in patients with and without irritable bowel syndrome (IBS)

Macronutrient or food group	No IBS *n* = 70	IBS *n* = 27	*p*-value
Carbohydrate (% of total energy intake)	43 (6)	45 (10)	0.20
Sugar (% of total energy intake)	5 (1–14)	5 (1–56)	0.34^#^
Protein (% of total energy intake)	19 (3)	17 (4)	0.04
Fat (% of total energy intake)	35 (6)	35 (9)	0.94
Saturated fat (% of total energy intake)	12 (2)	13 (4)	0.46
Monounsaturated fat (% of total energy intake)	12 (3)	12 (3)	0.95
Polyunsaturated fat (% of total energy intake)	7 (2)	6 (2)	0.52
Dietary fiber intake (intake in g/day)	33 (11)	32 (9)	0.53
Bread (intake in g/day)	176 (74)	175 (80)	0.99
Other cereals (intake in g/day)	54 (42)	70 (62)	0.14
Cakes (intake in g/day)	27 (44)	22 (24)	0.59
Potatoes (intake in g/day)	67 (48)	67 (51)	0.94
Vegetables (intake in g/day)	345 (197)	301 (166)	0.31
Fruit and berries (intake in g/day)	320 (251)	295 (188)	0.64

The results are given as mean (SD) for continuous variables with normal distribution and median (range) for other continuous variables. Energy intake and intake of different food groups are estimated from food frequency questionnaires in a subset of 97 patients recruited at Innlandet Hospital Trust Gjøvik. Differences between patients with and without IBS are analyzed with t-tests and Mann-Whitney U test (marked with ^#^)

## Discussion

The prevalence of IBS in patients with morbid obesity varied significantly between the two centers. At OUH-A, the prevalence (8%) was comparable with that in the general population [1, 7]. At IHT-G, the prevalence (27%) was three-fold that in the general population from the same region [14]. A high prevalence of IBS is consistent with most other reports from obesity centres [8–10].

High serum LDL levels and self-reported psychiatric disorders were independent predictors of IBS. An association between LDL and IBS has been reported in some, but not all earlier studies [22, 23]. Dietary differences or altered fat absorption or metabolism are possible explanations for the association between IBS and high LDL.

Dietary differences can influence on IBS symptoms [24, 25]. High LDL levels can be considered as a biomarker of a diet rich in saturated fats and low in fibre [26]. A difference in diet is a probable reason for higher levels of LDL in the subjects with IBS. Dietary registrations on a subset of the patients give support to this hypothesis, with correlations between the intakes of saturated and monounsaturated fats and LDL. Subjects with and without IBS ingested comparable amounts of carbohydrates, fibre, grains and vegetables.

Altered fat absorption in patients with IBS, possibly associated with local low-grade inflammation in the gut or alteration of the gut microbiome [3, 27] could also explain raised LDL levels. Altered fat metabolism is a third explanation. Blood lipoprotein levels are mainly regulated by the hepatocytes. Non-alcoholic fatty liver disease, which could influence the function of hepatocytes, is strongly related to obesity and has been discussed in relation to IBS [8, 28, 29]. Data on fatty liver disease were not available.

The association between IBS and self-reported psychiatric disorders is in accordance with studies in patients with morbid obesity and in the general population [7, 8, 14], patients with morbid obesity are known to have higher levels of stress, anxiety, and depression [15]. Associations between IBS and vitamin B_6_ deficiency [18, 19] and low-grade systemic inflammation measured as CRP [16, 17] were not seen in this study.

The difference in the prevalence rates of IBS at the two centres rendered post hoc examinations desirable. Hemoglobin, bilirubin, cholesterol, LDL, TSH and free T4 all showed statistically significant differences between patients with and without IBS at IHT-G, but not at OUH-A. In addition, the comparisons of patients with IBS at the two centres also revealed differences in the thyroid function (Table 3). It is unlikely that accidental circumstances or small sample sizes explain these findings. Minor differences in the analyses at the local laboratories could in part explain the differences between the patients at the two centres (Table 1), but not the differences between patients with and without IBS at each centre. It, therefore, seems to be true differences between the patients at the two hospitals, in particular among the patients with IBS. OUH-A had a long tradition for bariatric surgery and recruited patients from an urban region, whereas IHT-G was a new center for bariatric surgery in a rural region. The patients at a new centre for bariatric surgery will probably differ from patients seen at a centre with long traditions. Different health care and screening of the patients in the urban region may influence the presence of comorbidity and lifestyle (e.g. IBS, thyroid dysfunction, unhealthy diet) when evaluated at the center. Dietary differences with an unhealthy fatty diet in the rural area might have contributed to the differences in lipid values and IBS. Hypothyroidism can lead to gastrointestinal symptoms including abdominal pain [30], and thyroid dysfunction might have contributed to the high prevalence of IBS at IHT-G. This study indicated that changes in the lipid metabolism and thyroid dysfunction might be poorly recognized causes of IBS in general and in patients with morbid obesity in particular. These findings could in part explain the differences between the study centres. Differences in prevalence rates of functional gastrointestinal disorders in patients recruited from different types of secondary clinics (gastroenterological or obesity clinics) have recently been highlighted by Bouchoucha et al. [11] The current study shows that large differences also exist between clinics of the same type (two obesity clinics).

Abdominal pain is common after bariatric surgery [31], and the clinical evaluation usually focuses on surgical complications. The current research on IBS in patients with morbid obesity indicate that IBS is an important cause of abdominal pain before bariatric surgery, and probably remains so after surgery [32, 33]. Risk factors of IBS identified before surgery may also be important after surgery.

This study is in agreement with other studies showing widely different prevalence rates of gastrointestinal comorbidities among patients referred to different obesity centres [8–12]. The observations indicate that the diet could be a modifiable risk factor of IBS in this group of patients. The high prevalence of IBS is relevant for the clinical care of patients with morbid obesity, and the differences in the prevalence rates between centres before surgery are of importance for the evaluation of abdominal pain and discomfort in different cohorts after bariatric surgery.

### Strengths and limitations

Both centres used a validated Norwegian translation of the Rome III questionnaire in similar clinical settings. The study population was judged as representative for the subjects referred to the clinics during the study period. The presence of the study nurse only 3 days per week at IHT-G did not reduce the representativeness.

The difference in the prevalence rates and the different size of the study population at the two centres exclude a valid and generalised conclusion about the prevalence of IBS in subjects referred for bariatric surgery. A possible contributing explanation for the high prevalence rate of IBS at IHT-G could be that before filling in the Rome III questionnaire, the subjects were asked about food intolerance and food related abdominal symptoms, which could have induced a recall bias and report of more abdominal discomfort.

The comprehensive evaluation of the patients strengthened the possibility to detect predictors of IBS and differences between the centres, but also increased the risk of type I errors. Other and more precise predictors could have strengthened the study further. Dietary registrations were done only in a subset of the subjects patients and did not contain information about fermentable oligosacchardies, disaccharides, monosacchardies and polyols (FODMAPs), and inflammation markers were restricted to CRP. Also, a more precise diagnosis of the psychiatric disorders had been desirable. The blood tests were analysed at the local laboratories which limited comparisons between the centres, but did not affect the comparisons between patients with and without IBS at each centre and was adjusted for in the multivariate analyses (Table 2).

## Conclusions

The prevalence of IBS varied threefold between the two study centres. High LDL in serum and self-reported psychiatric disorders were predictors of IBS. Thyroid dysfunction might have contributed to the observed differences between the centres. A high intake of saturated fat and thyroid dysfunction could be modifiable risk factors of IBS, and attention to IBS is important in the care of patients with morbid obesity.

## Abbreviations

BMI: Body mass index
CI: Confidence interval
CRP: C-reactive protein
IBS: Irritable bowel syndrome;
IHT-G: Innlandet Hospital Trust Gjøvik
LDL: Low-density lipoprotein
OUH-A: Oslo university hospital Aker
T_4_: Free thyroxin
TSH: Thyroid stimulating hormone
